# Effect of Shot Peening on the Low-Cycle Fatigue Behavior of an AA2519-T62 Friction-Stir-Welded Butt Joint

**DOI:** 10.3390/ma16227131

**Published:** 2023-11-11

**Authors:** Robert Kosturek, Tomasz Ślęzak, Janusz Torzewski, Magdalena Bucior, Władysław Zielecki, Lucjan Śnieżek, Jarosław Sęp

**Affiliations:** 1 Faculty of Mechanical Engineering, Military University of Technology, 2 Gen. S. Kaliskiego Str., 00-908 Warsaw, Poland; tomasz.slezak@wat.edu.pl (T.Ś.); janusz.torzewski@wat.edu.pl (J.T.); lucjan.sniezek@wat.edu.pl (L.Ś.); 2 Department of Manufacturing Processes and Production Engineering, Rzeszow University of Technology, 8 Powstańców Warszawy Str., 35-959 Rzeszow, Poland; magdabucior@prz.edu.pl (M.B.); wzktmiop@prz.edu.pl (W.Z.); jsztmiop@prz.edu.pl (J.S.)

**Keywords:** friction stir welding, aluminum, shot peening, mechanical properties, fatigue, fracture

## Abstract

In this investigation, an AA2519-T62 FSW butt joint was subjected to shot peening with an air pressure of p = 0.6 MPa, a processing time of t = 10 min (per side), and a steel ball diameter of d_k_ = 1.5 mm. In order to evaluate the impact of shot peening on the low-cycle behavior, the samples were tested with coefficient R = 0.1 at total strain amplitudes of 0.35%, 0.4%, and 0.5%. The shot-peened welds are characterized by a higher value of stress amplitude, a lower value of plastic strain amplitude, and their fatigue life increased slightly. The cyclic strength coefficient and the cyclic strain hardening exponent were reduced by 45% and 55%, respectively, as the result of the surface layer hardening. The shot peening process had no noticeable effect on the character of crack initiation and propagation. Almost in all cases, the cracking started in the area under the weld face, located close to the boundary between the thermo-mechanically affected zone and the stir zone at the advancing side. Only at the heaviest loadings (ε_ac_ = 0.5%) were cracks initiated in the heat-affected zone at the retreating side. Despite the introduction of small cracks in the stir zone, their presence did not affect the decohesion character of the welded joint. Overall, it was observed that there is a minimal, positive impact of shot peening on the properties of the investigated joints.

## 1. Introduction

Friction stir welding (FSW) is a highly effective technique for joining precipitation-hardened aluminum alloys, also providing good mechanical properties for the obtained joints in conditions of cyclic loading [1,2,3]. In recent years, many efforts focused on increasing the fatigue performance of the FSW joints, mostly concerning 2XXX aluminum alloys [4,5,6,7]. The first solution involves, predominantly, a reduction in heat at the stage of the welding process by increasing tool traverse velocity or applying additional cooling, which limits the unfavorable heat-activated evolutions of strengthening phases (dissolution, overaging, etc.) [1,5,8]. Generally, the increase in welding velocity produces positive effects, e.g., a decrease in plastic strain amplitude in low-cycle fatigue (LCF) testing, as has been reported by Xu et al. [1]. A similar outcome can be achieved by applying additional cooling (e.g., water, liquid nitrogen, or carbon dioxide) directly after the passage of the welding tool [1,4,8]. A comprehensive review of the so-called rapid cooling techniques in friction stir welding and processing was written by Iwaszko [9].

The second solution is based on post-weld processing, mainly surface cold-working processes [10,11,12]. It has to be mentioned that, in conventional welding of precipitation-hardened aluminum alloys, post-weld heat treatment is the most efficient way of improving the mechanical properties of welded joints, but in the case of the FSW joints it gives a highly unfavorable coarse grainy structure due to abnormal grain growth, reducing overall fatigue performance [13]. Although some researchers have managed to avoid abnormal grain growth by modification of the heat treatment parameters [14]. In terms of FSW joint surface treatment, at the current state of the art, there are investigations focused on shot peening, laser shock peening, and brushing [10,12,15]. These types of treatments reduce surface roughness [15], introduce compressive residual stress, and increase the hardness of a surface layer [10,12]. Considering the fatigue properties of FSW joints, the surface treatment affects the high-cycle fatigue performance, since the condition of a surface and residual stress are the crucial factors determining fatigue life [16].

Ali et al. investigated the fatigue properties of a shot-peened 2024-T3 FSW joint and reported that the enhanced fatigue life is the result of slow near-surface crack propagation, possible due to the simultaneous application of increased microhardness and compressive residual stresses [11]. A reduction in the fatigue crack growth rate was also stated by Hatamleh et al., when investigating the impact of laser shock peening on the fatigue performance of friction stir-welded AA7075-T7351 [12]. Although the effects of surface treatment on the high-cycle fatigue properties of aluminum alloys have been described, its impact on low-cycle fatigue (LCF) properties is a gap in the current state of the art. In the LCF regime, it is microstructure factors that play a predominant role in fatigue performance [17]. Surface treatment can have a beneficial impact on the fatigue properties of high-strength aluminum alloys, which has been proven, for example, by Pandey et al., in their investigation on ultrasonic shot peening of the AA7075 alloy [18]. The reported increase in fatigue performance was due to the combined beneficial effects of grain refinement in the surface region and the associated compressive stresses without damage to the treated surface [18]. The potential of increasing fatigue performance is not only limited to the base material but can be applied to FSW joints. Nowadays, the improvement in performance of armor grade aluminum alloy FSW joints includes a.o. studies on stationary shoulder FSW (SSFSW) and underwater FSW (UWFSW) [19,20,21]. 

Analyzing the current state of the art, the conclusion can be drawn that post-processing of the FSW joints is a major gap from a scientific point of view. In many cases, despite providing data on, for example, microstructure and residual stress, papers rarely address the fatigue properties. In the previous research on the FSW of Sc-modified AA2519-T62 extrusion, the authors investigated the impact of welding parameters on its LCF behavior [2] and the role of the low-hardness zone (LHZ) in LCF failure [22]. As the continuation of this research, the authors aim to investigate the effect of post-weld processing in the form of shot peening on LCF behavior of the considered FSW joints. In this investigation, the focus is on the LCF, which not only is characterized by a significantly lower lifetime of elements (up to 20,000 cycles) but also, due to the presence of plastic deformation in each cycle, the effect of surface condition and residual stress play a secondary role in favor of the microstructure. The chosen approach aims to evaluate how the proposed surface treatment can influence the welded elements subjected to cyclic, heavy loading, which takes place, for example, in aircraft components.

## 2. Materials and Methods

The investigated material was 5 mm-thick AA2519-T62 extrusion (IMN Oddział Metali Lekkich, Skawina, Poland). The chemical composition and mechanical properties are shown in Table 1 and Table 2, respectively. 

The alloy was welded via the FSW method using an ESAB Legio 4UT machine (ESAB, Göteborg, Sweden) and following welding parameters: 600 rpm tool rotation speed, 100 mm/min tool welding velocity, 2° tool tilt angle, and MX Triflute tool (ESAB, Göteborg, Sweden) dedicated for 5 mm thick workpieces. The welding parameters were set based on previous research performed by the authors [2]. The basic mechanical properties of the welded plate are shown in Table 3.

**Figure 1:**
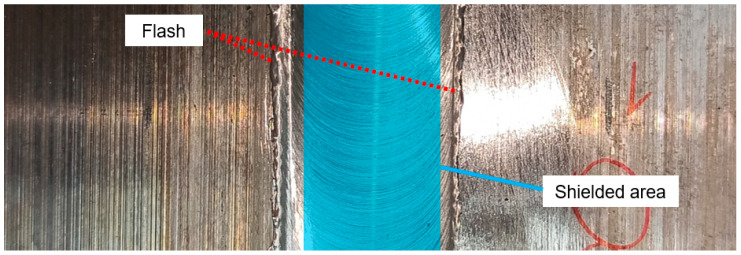
Weld face with marked shielded area.

After the welding, the obtained joint was subjected to pneumatic shot peening. The peening was applied only to the heat-affected zone and the thermo-mechanically affected zone of the weld face using special plastic covers (omitting the stir zone) according to the scheme in Figure 1. 

**Figure 2:**
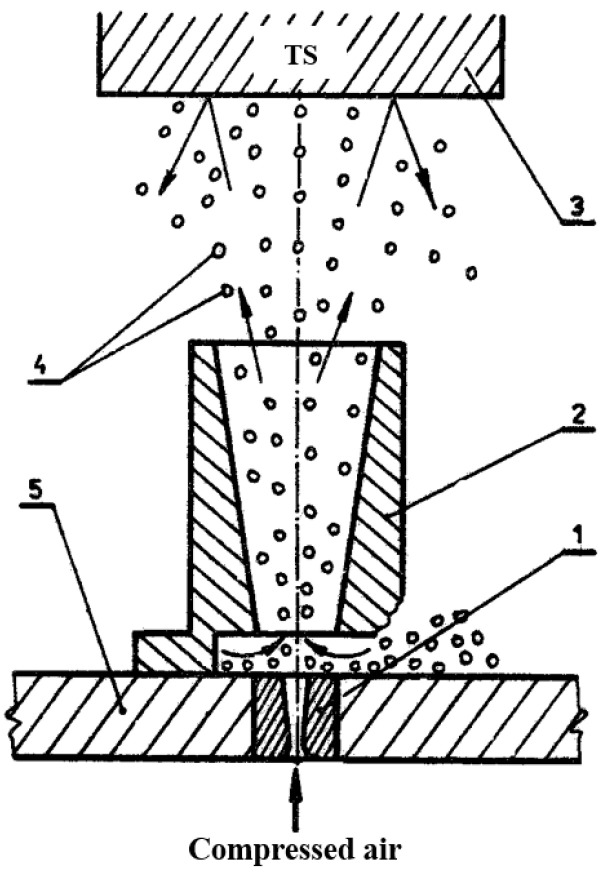
Scheme of the shot peening process: bottom nozzle (1), upper nozzle (2), treated surface (3), steel balls (4), bottom of work chamber (5).

Before the shot peening, the flash (marked in Figure 1) was mechanically grounded to obtain a smooth upper surface. The following process parameters were used: air pressure p = 0.6 MPa, processing time t = 10 min (per side), steel ball diameter d_k_ = 1.5 mm. The parameters were proposed based on the mechanical properties of the treated alloy. The scheme of the shot peening process is presented below (Figure 2).

**Figure 3:**
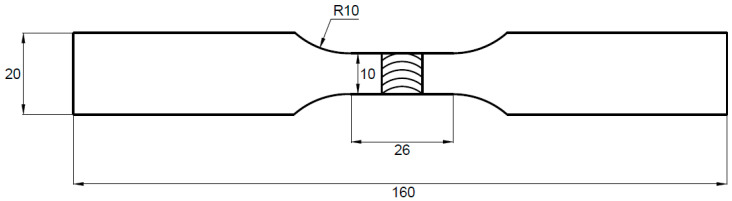
Scheme of the fatigue sample.

The lower nozzle (de Laval nozzle) was supplied with compressed air at a pressure of 0.6 MPa. The airflow stream exiting from the lower nozzle entrained steel balls from the bottom of the working chamber and then directed them through the upper nozzle onto the processed sample, attached to the inner side of the cover. The distance between the processed surface and the upper nozzle was 100 mm. The intensity of peening was determined based on the deflection arrow of control plates and is expressed in Almen units (corresponding to the deflection arrow value of the control plate expressed in hundredths of an inch or mm). After the peening of welded joints, the deflection arrow of type A control plates was 0.15 mm. The samples in the as-welded state and after shot peening were investigated in terms of macrostructure, and low-cycle fatigue properties supported by fractography analysis. The macrostructure of the metallographic specimen was revealed using Keller reagent (20 mL H_2_O + 5 mL HNO_3_ + 1 mL HCl + one drop of HF) with an etching time of 10 s. The macro- and microstructure observations were conducted on an Olympus LEXT OLS 4100 digital light microscope. The LCF tests were carried out on specimens with a gauge width of 10 mm and length of 25 mm according to ISO standard 12106:2017 in a total strain-controlled condition with asymmetry coefficient R = 0.1 [23]. The applied total strain amplitudes were: 0.35%, 0.4%, 0.5%. The scheme of the fatigue sample is presented in Figure 3.

An axial clip extensometer with a gauge length of 25 mm was used to control the applied strain. The fatigue failure criteria were defined when the maximum tensile stress dropped by 20% below that at initial life. The fracture surfaces of failed fatigue samples were examined using a Jeol JSM-6610 scanning electron microscope.

## 3. Results and Discussion

In order to evaluate the impact of the shot peening process on the properties of the welded joint, macrostructural observations and fatigue testing were performed.

### 3.1. Macrostructural Observations

**Figure 4:**
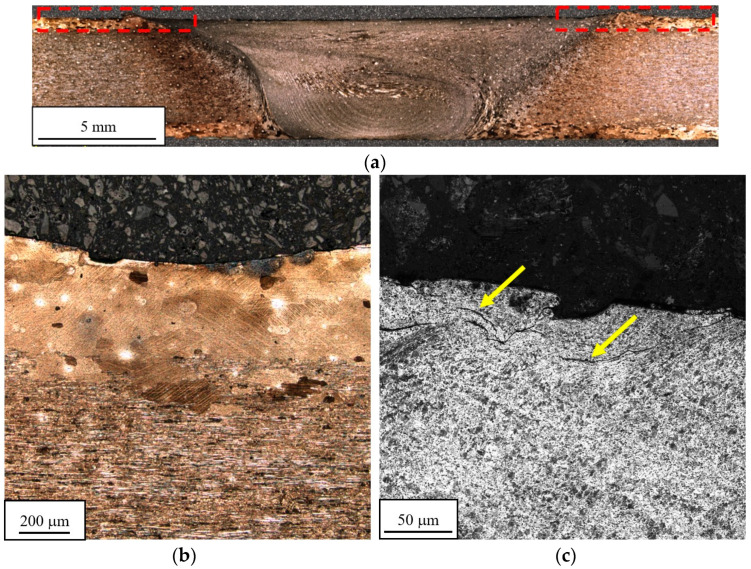
FSW joint after shot peening: macrostructure (**a**), heat-affected zone (**b**), shoulder-driven zone (**c**).

The first stage of the research involved macro- and microstructural analysis of treated welded joints. This study focused solely on the impact of the peening process on the structure of the welded joints, rather than on the structure of the joints in the as-welded state, as has been described in previous works [2,22]. The macrostructural and microstructural images of the welded joint after shot peening are presented below in Figure 4.

The area subjected to shot peening (highlighted in red) corresponded to the upper part of the heat-affected and thermo-mechanically affected zones (Figure 4a). Beneficial deformation of large near-surface grains was observed (Figure 4b). This layer of coarse grains occurs in extruded heat-treated aluminum alloys, and in the previous investigation [22] was identified as a factor promoting the initiation of fatigue cracks in the low hardness zone. Simultaneously, the impact of steel balls led to the unfavorable introduction of small cracks in the ultrafine-grained part of the welded joint at a depth of about 30 µm (Figure 4c). The cracks appeared in the section of the welded zone which was not shielded by the plastic cover. In order to confirm the effect of the proposed surface treatment, three different sets of samples were subjected to the shot peening, and in every case the cracks appeared in the discussed area. At the same time, no presence of cracks has been reported in the relatively highly deformed overgrowth grains in the HAZ (Figure 4b). From the microstructural point of view there is small, positive in the form of HAZ upper layer plastic deformation.

### 3.2. Low-Cycle Fatigue Properties

**Figure 5:**
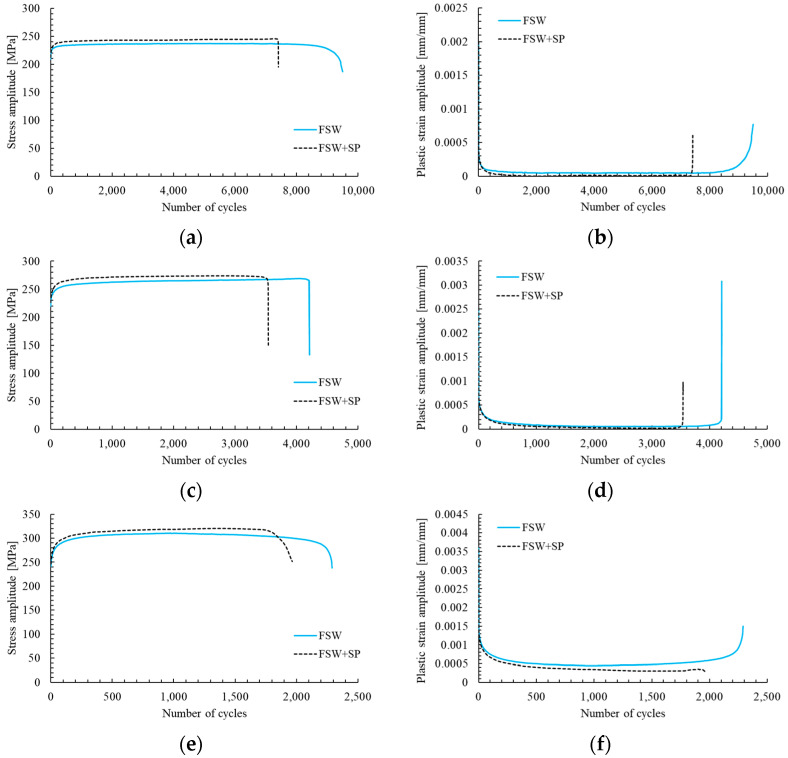
Stress (**a**,**c**,**d**) and plastic strain amplitudes (**b**,**d**,**f**) vs. the number of cycles for the as-welded samples (FSW) and shot-peened joints (FSW + SP) at total strain amplitude of ε_ac_ =0.35% (**a**,**b**), ε_ac_ =0.4% (**c**,**d**) and ε_ac_ = 0.5% (**e**,**f**).

The curves presenting changes in stress amplitude and plastic strain amplitude in the number of cycles for selected samples are presented below in Figure 5a–f.

**Figure 6:**
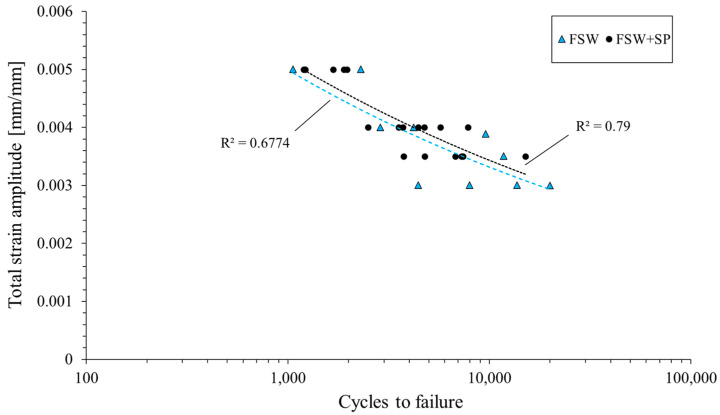
Low-cycle fatigue life curves.

The obtained curves allow us to draw the conclusion that the both as-welded and shot-peened samples have three stages of fatigue life: a short, initial period of cyclic hardening, then a long period of stable cyclic properties, and finally, cyclic softening until failure. In the performed investigation, the welded joints subjected to the shot peening are characterized by a higher value of stress amplitude and a lower value of plastic strain amplitude. These results overlap with other studies on the low-cycle fatigue of shot-peened samples [22,24]. The cycle fatigue curves are presented in Figure 6. Generally, the obtained data suggest that the shot peening process slightly increased the fatigue life of the FSW joints in the investigated range of total strain amplitudes. Additionally, the peened samples are characterized by a higher repeatability, which is reflected in the values of the R^2^ coefficients.

**Figure 7:**
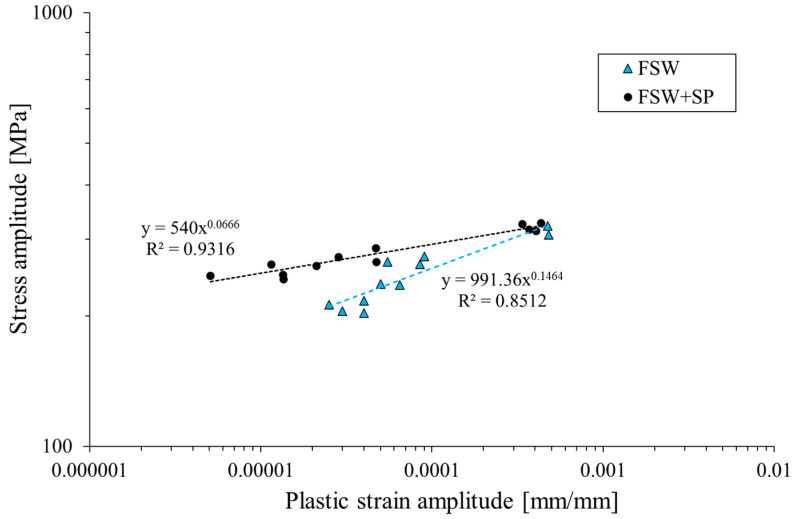
Cyclic stress–strain curves in log–log coordinates.

The stabilized cyclic parameters were used to establish a relationship between stress and plastic strain amplitude for the two investigated conditions of the welded joint (Figure 7).

**Figure 8:**
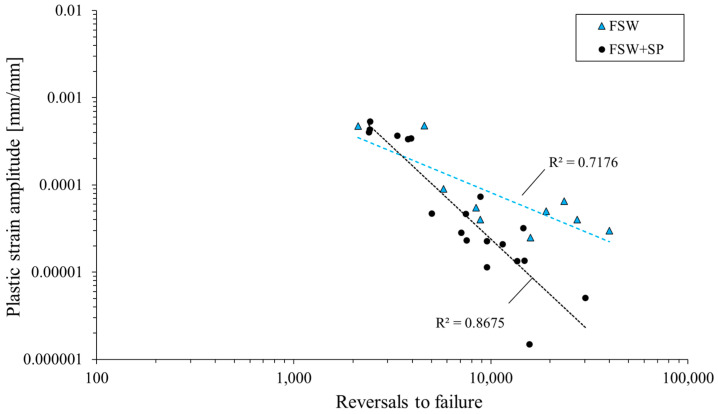
Plastic strain amplitude vs. reversals to failure.

The curve in log–log coordinates can be described by the following equation [25]:

*σ_a_* = *K′*(*ε_ap_*)*^n′^*

where *K′* is the cyclic strength coefficient and *n′* is the cyclic strain hardening exponent. The analysis of the obtained curves allows us to state that the shot peening process influenced the fatigue properties of the welded joint. Both the cyclic strength coefficient and the cyclic strain hardening exponent were reduced by 45% and 55%, respectively, as the result of surface layer hardening. This feature can be also observed in the registered values of plastic strain amplitude in the function of reversals to failure, presented in Figure 8.

Analysis of the obtained plot shown allows to draw the conclusion that shot-peened samples have lower values of plastic strain amplitude than as-welded samples in the condition of cyclic loading. These differences are only visible for relatively low levels of the tested strain amplitudes (ε_ac_ = 0.35%), and together with an increase in strain level, the positive effect of shot peening is difficult to observe. Overall, the applied surface treatment has allowed for a minimal increase in the fatigue life of FSW joints (Figure 6) and a more distinct cyclic hardening in the initial stage of fatigue testing (Figure 5). In this regard, the obtained results align with those presented in the study on ultrasonic shot peening of the AA7075 alloy by Pandey et al. [18]. The lower capacity for cyclic hardening, reflected in the plastic strain amplitude (Figure 5b,d,f) can be connected with the plastic deformation of the HAZ overgrowth grain layer (Figure 4b) during the shot peening. This results in the layer’s lower potential for further strain hardening in LCF testing, but only in the case of ε_ac_ = 0.35%. For higher total strain amplitudes, the behavior of the samples is very similar and, in both cases, a significant cyclic hardening occurs.

### 3.3. Fracture Behavior

**Figure 9:**
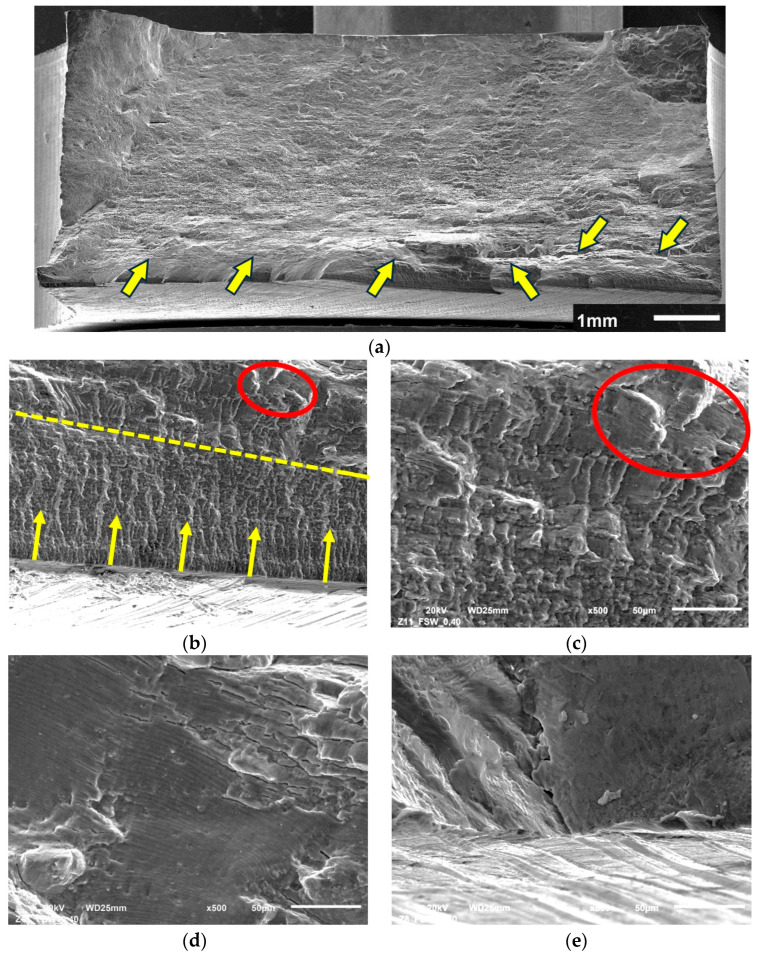
Fracture surface of FSW joints subjected to LCF tests: sample tested at ε_ac_ = 0.4% (**a**–**d**) and sample tested at ε_ac_ = 0.5% (**e**); description in text.

**Figure 10:**
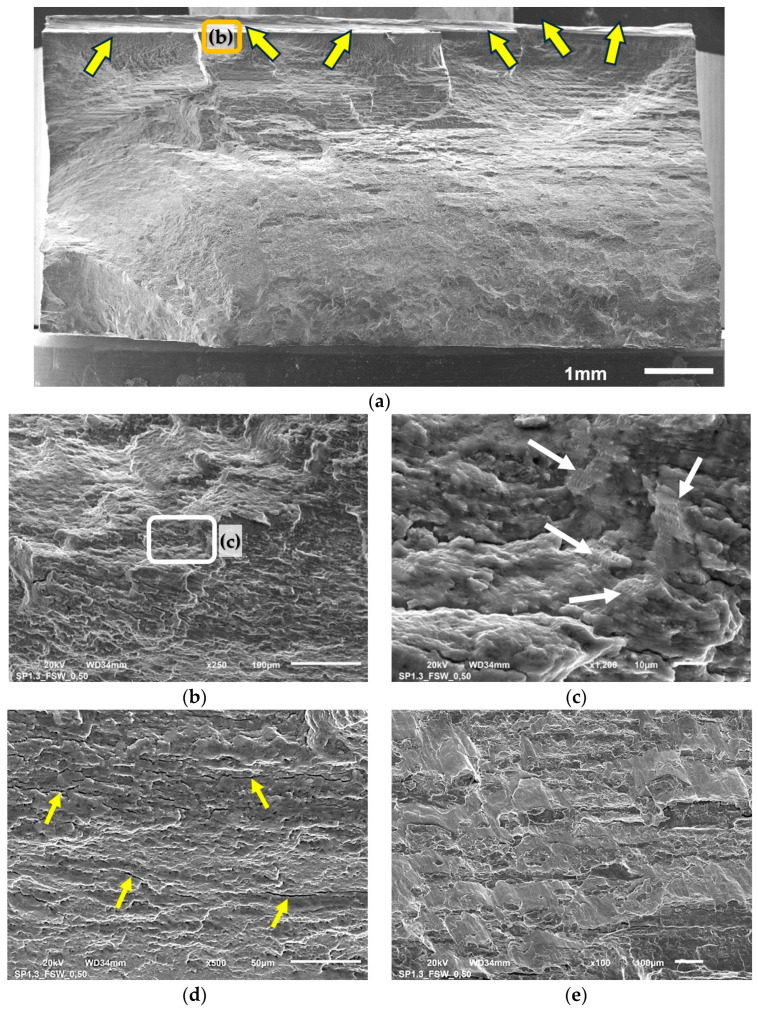
Fracture surface of the shot-peened FSW joint of a sample subjected to LCF tests at ε_ac_ = 0.4%; overall view (**a**), faster crack growth zone (**b**,**c**), secondary cracks (**d**), very high crack propagation zone (**e**); description in text.

The vast majority of samples subjected to fatigue tests cracked on the advancing side of the joint in the area affected by the edge of the tool’s shoulder, which corresponds to the TMAZ/SZ interface. In both cases, before and after shoot peening, the samples loaded under ε_ac_ = 0.5% cracked at the retreating side out of the stirred region, in the heat-affected zone. Selected samples were subjected to fractographic analysis using SEM and the results are presented in Figure 9 and Figure 10.

A general view of the fatigue fracture surface of the sample before shot peening is shown in Figure 9. The cracks were initiated simultaneously with many origins starting from surface defects and they are marked with arrows in Figure 9a. Then, as they grew, they merged with transverse cracks into larger ones, which caused the crack to grow faster.

In Figure 9b the near-to-surface crack zone can be observed as the crack propagates slowly until it has reached the yellow dashed line. The crack propagation rate then accelerated after exceeding it. In this zone, the fatigue striations can be observed, which are circled in red (Figure 9b,c). Exemplary fatigue striations observed in the high-crack-propagation-rate zone are shown in Figure 9d. What is more, there were also secondary cracks courses observed, which, in the case of high loading rate, is related to surface line defects (Figure 9e). Similarly, a localized net of secondary cracks was also reported by Trško et al. in fatigue testing of shot-peened AA7075 [26]. Shot peening did not influence the nature of crack initiation because, similarly to not-shot-peened samples, the cracks were initiated simultaneously in many places (Figure 10a). The regions of stable fatigue crack growth are indicated with the arrows. The selected region is shown in Figure 10b,c and analyzed in detail.

This region is the zone of faster crack growth. In this area, the material is mostly shearing (Figure 10b) and small fatigue striations can be observed under a higher magnification (Figure 10c). A heavy load has caused the secondary cracks to develop (Figure 10d) but they were not related to surface defects like in the case shown in Figure 9e. The zone of the very high crack propagation stage is shown in Figure 10e. In all cases, the dominant failure mechanism is typical for precipitation-hardened aluminum alloys microvoid coalescence (MVC), with the voids forming at small inclusions of the secondary phase giving the characteristic, dimple structure. The vast majority of the surface was sheared ductile and strongly deformed during the compressive stage of the load.

The additional conclusion is that despite the presence of small cracks in the stir zone (Figure 4c) introduced by the shot penning process, there is no evidence of there being any effect on the decohesion of the tested joints. The failures occurred at the boundary between TMAZ and SZ, typically for precipitation-hardened aluminum alloy FSW joints [1,2]. Due to its fine-grained microstructure, the SZ is characterized by the low value of the fatigue crack growth rate [27]. Additionally, the compressive residual stress also contributes to the crack closure effect in the stir zone [27]. The introduction of microcracks by the shot peening can be avoided via optimization of the process parameters or through the use of an additional protective element for the ultrafine-grain area. Although, it has to be taken into consideration that, based on the obtained results, shot peening produces a relatively small, positive effect in terms of the AA2519 FSW joint’s LCF properties, predominantly for low values of strain amplitude (0.35%). Surface treatment influences, in the first place, the crack initiation period of fatigue life, but no noticeable effect on near-surface fracture behavior has been noticed.

## 4. Conclusions

On the basis of the obtained results the following conclusions can be stated:

1. The performed shot peening process caused the plastic deformation of large near-surface grains, but also introduced small microcracks into the stir zone at a depth of about 30 µm.
2. The shot-peened welds are characterized by a higher value of stress amplitude, a lower value of plastic strain amplitude, and their fatigue life slightly increased. The cyclic strength coefficient and the cyclic strain hardening exponent were reduced by 45% and 55%, respectively, as the result of the surface layer hardening.
3. The shot peening process had no noticeable effect on the character of crack initiation and propagation. Almost in all cases, the cracking started in the area under the weld face, located close to the boundary between the thermo-mechanically affected zone and the stir zone at the advancing side. Only in the heaviest loads (ε_ac_ = 0.5%) were the cracks initiated out of the FSW region at the retreating side.
4. Despite the introduction of small cracks into the stir zone, their presence did not affect the decohesion character of the welded joint with failure occurring in the thermo-mechanically affected zone at the advancing side.

Considering the obtained results, it can be concluded that performing the shot peening process for a 5 mm thick AA2519-T62 FSW butt joint may not be necessary if the welded element is intended for operating in the LCF regime, for example, as an aircraft component.

## Figures and Tables

**Table 1:** Chemical composition (wt.%) of the AA2519 alloy.

Si	Fe	Cu	Mn	Mg	Ni	Zn	Ti	Zr	Sc	V	Al
0.08	0.11	6.32	0.17	0.33	0.02	0.05	0.008	0.19	0.16	0.10	Base

**Table 2:** Basic mechanical properties of the AA2519 alloy.

Young Modulus, E	Yield Strength, R_0.2_	Tensile Strength, R_m_	Elongation, A
78 GPa	312 MPa	469 MPa	19%

**Table 3:** Basic mechanical properties of the AA2519 friction-stir-welded joint.

Young Modulus, E	Yield Strength, R_0.2_	Tensile Strength, R_m_	Elongation, A
72 GPa	265 MPa	410 MPa	9%

## Data Availability

Data available on request.
